# Investigating the Effects of Threatening Language, Message Framing, and Reactance in Opt-Out Organ Donation Campaigns

**DOI:** 10.1093/abm/kaab017

**Published:** 2021-05-03

**Authors:** Jordan Miller, Lesley McGregor, Sinéad Currie, Ronan E O’Carroll

**Affiliations:** Division of Psychology, Faculty of Natural Sciences, University of Stirling, Stirling FK9 4LA, UK

**Keywords:** Organ donation, Opt-out consent, Message framing, Threat to freedom, Reactance

## Abstract

**Background:**

Under opt-out organ donation policies, individuals are automatically considered to have agreed to donate their organs in the absence of a recorded opt-out decision. Growing evidence suggests that the language used within organ donation campaigns influences donor intentions and decision-making.

**Purpose:**

As awareness campaigns to promote opt-out consent in the UK are ongoing, the objectives of this study were to investigate the effect of language and message framing used in opt-out organ donation campaigns on donor intentions and psychological reactance.

**Methods:**

Individuals from Scotland and England (*N* = 1,350) completed this online experiment. Participants were randomized to view one of four messages, designed in the format of a newspaper article, which described the upcoming opt-out system. This followed a 2 × 2 design whereby the degree of threatening language (high threat vs. low threat) and message framing (loss vs. gain) of the newspaper article was experimentally manipulated. Measures of intention (pre-exposure and postexposure) and postmessage reactance (threat to freedom and anger and counter-arguing) were obtained.

**Results:**

A mixed analysis of variance revealed a significant Group × Time interaction on donor intentions; post hoc analysis revealed that intentions significantly decreased for individuals exposed to the High threat × Loss frame article but significantly increased for those exposed to the High threat × Gain frame article.

**Conclusions:**

In campaigns to promote opt-out legislation, high-threat language combined with loss-frame messages should be avoided. If high-threat language is used, gain-frame messaging that highlights the benefits of organ donation should also be incorporated.

## Teaser

Within opt-out campaigns, high-threat language and loss-frame messaging decreased donor intentions. Emphasizing the benefits of donation using gain-frame messaging, within campaigns that contain high threat language, increased intentions.

## Introduction

Across the world, there is a disparity between the limited number of organ donors and the growing demand for transplantation [1]. In an effort to increase the number of donors, nations across the world are implementing opt-out consent legislation. In the UK, this legislation, which was implemented in Wales in 2015, England in May 2020, and is planned for Scotland in March 2021, removes the requirement for active registration on the national donor register to indicate consent for organ donation [2]. Instead, eligible adults will be automatically considered to consent for organ donation in the absence of a formally recorded opt-out decision.

Consistently, evidence has emphasized the important role of affective beliefs (e.g., medical mistrust) on donor decision-making among nations with opt-in organ donation policies [3–5]. However, few studies have investigated the possible factors influencing donor decision-making under opt-out consent. Research from other nations with opt-out legislation has suggested that there may be specific factors associated with the legislative change that drive opt-out decisions. For example, the opt-out policies in Brazil and Chile were revised following a considerable postimplementation decline in transplantation rates and an increase in family refusal [6–8]. This was attributed to heightened concerns of medical mistrust and general distrust in the government. Notions of unwarranted government control were also reported among members of the Welsh population preceding the introduction of opt-out consent [9]. Similar concerns have emerged within our recent qualitative research, which explored the reasons behind anticipated opt-out decisions in Scotland and England [10]. The results indicated that participants regarded opt-out consent as giving the government “ownership” over their body and donor decision. Throughout, participants emphasized the importance of autonomy regarding donor-relevant decisions and voiced frustrations at the notion of presumed consent, in turn, suggesting that this would threaten their “free will to make their own decisions.” Therefore, opting out of organ donation was viewed as a way of protecting one’s autonomy. As opt-out decisions appear to be driven by a desire to protect freedom of choice, the concept of psychological reactance may contribute to our understanding of these deterrents.

Psychological reactance is an aversive motivational response arising when an individual perceives their behavioral freedoms to be under threat [11]. In response, psychological reactance theory posits that individuals will be driven to take action to safeguard or reinstate control over the notion they perceive as being under threat [11, 12]. In broad terms, behavioral freedoms are a set of actions, values, or attitudes a person expects they should be able to enact without restriction or coercion from external sources [12, 13]. Accordingly, within the context of health decision-making, the choice to register or not to register as an organ donor can be categorized as a free behavior. This free behavior subsequently becomes more desirable when it is believed to be restricted or threatened [11].

Reactance is defined in this study in accordance with Dillard and Shen’s conceptualization: a combination of anger and counterarguing, which arises following exposure to a stimulus perceived to threaten one’s behavioral freedom [14]. This threat-based response drives restoration of freedom by influencing attitudes and behavioral intentions. Within the context of health communication, restoration of freedom often manifests in the form of an unintended “boomerang effect,” whereby reactant readers will engage in oppositional behaviors in response to health warnings, for example, increased alcohol consumption following exposure to alcohol warning messages [15, 16]. Thus, if information regarding opt-out consent arouses a sense of threat to freedom, reactance, which manifests as a composite of anger and counterarguments toward the source, may occur. This in turn, induces negative attitudes and may adversely impact behavioral intentions toward organ donation.

### The Role of Reactance in Health Decision-Making

Reactance is particularly important in the context of change situations, including political and health care reforms. This is partly attributable to the use of language within such communications, for example, persuasive terminology, such as “you must,” “you have to,” and “you need,” are often perceived as being more threatening and restrictive than autonomy-supportive language, such as “you may,” “you could,” and “consider” [17, 18]. Research across a variety of public health domains has investigated this, reporting the application of persuasive and high-threat language to induce perceptions of threat to freedom, heightened negative cognitive responses, and anger in comparison to low-threat messages [14, 17–20]. Given their often direct and persuasive nature, this may explain why public health campaigns and interventions to change health behaviors can incur undesirable consequences.

A small number of studies have applied this to the context of organ donation campaigns and reported the use of high-threat language to increase perceptions of threat to freedom, which, in turn, induced state reactance [21, 22]. However, the language manipulation employed in the following study was particularly authoritative and unlikely to be used in practice, for example, “Stop the denial! Given the need for organ donors, a reasonable person would consent to be an organ donor” [22]. Therefore, it is somewhat unsurprising that those exposed to the high-threat condition reported perceptions of restricted freedom and experienced reactance-related negative cognitions and anger. The application of such overtly forceful language may not be appropriate for use in health communication campaigns. As such, it is important to investigate whether more subtle manipulations, using language that is routinely used within the public domain, can induce a sense of restricted freedom and elicit reactance.

### Message Framing

Differences in message framing (loss vs. gain) can also have a substantial impact on one’s behavioral intentions. This effect is attributed to prospect theory [23]. In short, when making decisions, an individual considers the degree of risk associated with each choice. Specifically, the way a message is framed, for example, presenting the benefits of a particular decision (*gain frame*) influences one’s risk-related preferences and subsequent decision-making. Applying prospect theory to health-related decision-making, “risk” refers to the likelihood of an individual experiencing unpleasant outcomes as a consequence of a particular action. A considerable body of literature has examined health message framing and reports a differential effect of framing manipulations for specific categories of health behaviors [24–27]. Thus, gain-frame messages, which emphasize the *benefits* of engaging in certain behaviors, are effective at promoting protective or preventative behaviors [26]. Loss-frame appeals, which highlight the adverse consequences of *not* engaging in certain behaviors, are most effective at encouraging health detection or diagnostic behaviors [27]. However, the application of these findings is somewhat challenging within the domain of organ donation as the implications of the behavior (registering as an organ donor) do not personally benefit the individual who enacts the decision.

A number of studies have explored framing effects in the context of organ donation [28–31]. However, the evidence base appears inconsistent, with some studies reporting no differential effect of framing on donor attitudes or behavior [28, 31]. Conversely, other research has reported the use of gain-frame messages to increase one’s willingness to become a living kidney donor [29]. Evidence within the domain of posthumous organ donation offers support for these findings, reporting that gain-frame messages were perceived as more favorable and resulted in greater behavioral intentions toward deceased organ donation than messages that contained a loss-frame component [30]. The impact of message framing on psychological reactance was also considered within this study. Interestingly, loss-frame messages were found to increase perceptions of reactance, which, in turn, adversely affected attitudes and donor intentions. Reactance was found to mediate the relationship between message frame and subsequent message response. The authors reasoned that presenting organ donation messages within a loss-frame (e.g., illustrating how many people die because of the donor shortage) may have elicited a sense of guilt among readers and led to perceptions of the message as being covertly forcible. These findings warrant further testing of message framing within organ donation literature.

### The Current Study

A considerable body of evidence has shown that the content of communication campaigns plays an important role in the attitudes and behavioral intentions of the public. Research to date has focused on message content and language within campaigns designed to encourage donor registration. However, evaluating language and message framing arguably becomes more important in the context of opt-out organ donation as those who have not registered a donor decision are now presumed to consent for organ donation. At the time of conducting this study, opt-out consent had been enacted in Wales and was scheduled for implementation in England and Scotland in mid-2020 (opt-out consent was later postponed in Scotland due to the COVID-19 pandemic). As far as the authors are aware, no previous research has investigated the role of threatening language and message framing in relation to opt-out organ donation campaigns. The present study, therefore, employs a between-group design to examine the role of language (high vs. low threat) and message framing (loss vs. gain) used within opt-out organ donation campaigns on donor intentions and the development of psychological reactance. We hypothesized that (a) the opt-out campaign that contains high-threat language and a loss-frame component will decrease donor intentions in comparison to low-threat and gain-frame campaigns, (b) high-threat language will induce greater threat to freedom than campaigns that use low-threat language, and (c) participants who plan to opt-out of the donor register will exhibit higher levels of reactance (manifesting as greater threat to freedom, anger, and counter-arguing) when exposed to campaigns promoting opt-out consent.

## Methodology

### Participant Eligibility Criteria and Recruitment

Ethical approval for this study was obtained from the University of Stirling’s General University Ethics Panel. Recruitment took place between November 2019 and February 2020. During this time, neither England nor Scotland had introduced opt-out consent, though wide-spread campaigns to promote the legislative change were underway. As such, only adults (18 + years) living in England and Scotland were eligible to participate. Participants were opportunistically recruited for this study through UK-wide mailing lists and via social networking sites (Twitter and Facebook). The study link was also shared with academic colleagues across the UK. Advertisements were also placed on the University of Stirling Portal announcement page, which is used for publicizing research studies to students and staff. Lastly, recruitment posters with a study URL link and a QR code were displayed in various shops, bus stops, and faith centers around central Scotland. As an incentive, participants had the opportunity to enter a prize draw for a £25 Amazon voucher.

### Power Analysis

A G*Power calculation was conducted to determine the number of participants required to detect a small effect size of *f* = .10 in line with Cohen’s guidelines [32]. This indicated that using a between-group comparison with four groups at an alpha level of .05 and a power of .80, a sample size of 1,292 was required (approximately 320 in each of the four arms).

### Design

This study employed a 2 × 2 between-groups design, whereby the language threat level (high vs. low) and message framing (loss vs. gain) of opt-out organ donation campaign messages were experimentally manipulated. Newspaper and electronic articles are a key communication medium for information regarding organ donation; therefore, to enhance ecological validity, each message was designed in the format of a newspaper article. Participants were, therefore, randomly assigned to view one of four message conditions: *Condition 1:* Low threat × Gain frame; *Condition 2:* High threat × Gain frame; *Condition 3:* Low threat × Loss frame; *Condition 4:* High threat × Loss frame.

### Procedure

**Fig. 1.**
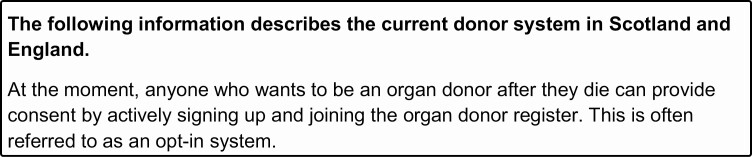
Information presented to participants to describe the existing opt-in system.

This study involved the completion of an online questionnaire hosted on Qualtrics, a web-based questionnaire platform. A diagram of the study procedure is available in Supplementary Information 1. Participants accessed the survey via an anonymous URL link or QR code affixed to the study recruitment posters. After informed consent was collected via an electronic checkbox at the beginning of the questionnaire, participants were presented with brief information describing the opt-in donor system that was, at the time of recruitment, operational in Scotland and England (see Fig. 1).

**Fig. 2.**
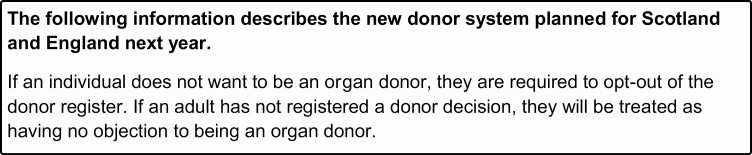
Information presented to participants to introduce the forthcoming opt-out system.

Baseline donor intentions were then recorded in response to the following statement “I intend to donate my organs after death.” To then assess planned donor status following the change in legislation, participants were presented with a description of the opt-out system (see Fig. 2) and asked to indicate their anticipated donor decision by selecting from one of the following response options: (a) opt-in, (b) take no action and follow deemed consent, (c) undecided, and (d) opt-out.

Following this, demographic information was collected. To encourage continued engagement with the study, directly before presentation of the experimental conditions, participants were presented with a message stating “In the next section you will be asked to read an article about organ donation. Please read this carefully, you’ll be asked a few questions afterwards.” Participants were then randomly allocated to view one of four messages: Condition 1: Low threat × Gain frame; Condition 2: High threat × Gain frame; Condition 3: Low threat × Loss frame; Condition 4: High threat × Loss frame. In an effort to ensure that participants read the information within the allocated message, a timer function was embedded into the survey, which delayed progression onto the next stage of the study until 50 s had elapsed, approximately the time required to read each message. The duration was calculated using an estimated reading time generator for the four conditions (Read-o-Meter; http://www.niram.org/read/).

### Opt-Out Organ Donation Message Content

**Fig. 3.**
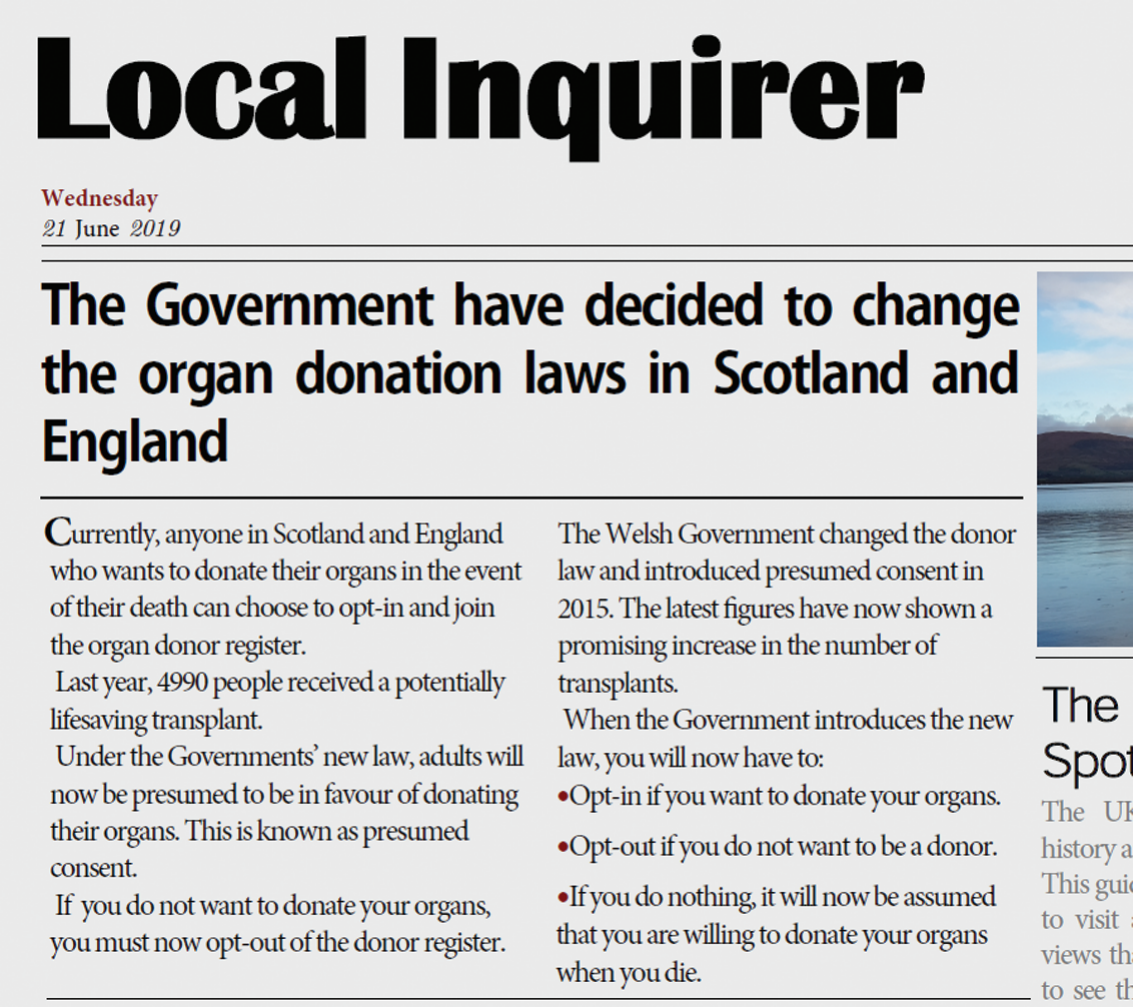
Example of Condition 2: High Threat × Gain Frame message.

As communication campaigns were underway in Scotland and England to ensure public awareness and understanding of the legislative change, each message was designed using Adobe Acrobat software to mimic a newspaper article (Fig. 3). The newspaper conditions were matched in content and contained a similar number of words (range: 164–180 words). The readability of the conditions was assessed using the Flesch–Kincaid Readability statistics and were deemed suitable to be read from approximately grade levels 7 and 8 [33]. Each message broadly contained information describing the current opt-in donor system, UK organ donation figures, the introduction of opt-out legislation, and the main donor decisions offered under opt-out consent.

The language threat manipulations were informed by existing reactance literature [14, 17, 18]. In addition, the manipulations were also informed by our recent qualitative study, which showed heightened reactance to arise following reference to opt-out legislation as a system of presumed consent [10]. Within the low-threat condition, neutral word choice and autonomy-supportive language was used throughout. For example, when describing donor decision-making under the opt-out system, the following phrase was used “If you decide you do not want to donate your organs, you can always choose to opt-out of the donor register.” Conversely, within the high-threat condition, the threat level was manipulated by the inclusion of more direct language, overtly forceful adverbs, and imperatives. Thus, when describing donor decision-making under opt-out consent, the reader was presented with a command that may restrict choice “If you do not want to donate your organs, you must now opt-out of the donor register.”

Two framing manipulations (loss vs. gain) were used in this study. The first framing manipulation presented figures on the number of people who had died on the waiting list (loss): “Last year, 400 people died while waiting for a potentially lifesaving transplant,” and how many people’s lives had potentially been saved by an organ transplant (gain): “Last year, 4990 people received a potentially lifesaving transplant.” The second framing manipulation centered around a description of the opt-out system in Wales. As Wales was the first UK nation to introduce opt-out consent, information on Welsh rates of donation following the legislative change feature heavily within UK opt-out media campaigns. Therefore, the gain-frame manipulation described the opt-out system in Wales to have resulted in “a promising increase in the number of transplants” and in the loss/neutral frame manipulation “a small increase in the number of transplants.” The specific message variants used in each of the language and framing manipulations are available in Supplementary Information 2.

Immediately after the presentation of the experimental conditions, participants completed a postintervention measure of donor intentions and three scales measuring the key aspects of reactance in response to the message: threat to freedom, anger, and counter-arguing. Participants then completed two further scales measuring the readability and credibility of the messages. The newspaper condition each participant had been allocated during randomization was displayed again prior to the completion of the readability and credibility scales.

### Primary Outcome Measures

Each of the primary outcome measures were scored using a seven-point Likert scale, ranging from 1 (*strongly disagree*) to 7 (*strongly agree*).

#### Donor intentions

The primary outcome measure, change in donor intentions, was measured at two time points (baseline and postmessage exposure). Participants were asked to respond to the following statement “I intend to donate my organs after death.” Higher scores indicate greater intentions to donate organs.

#### Threat to freedom

Participants completed a four-item validated measure of threat to freedom used extensively within reactance research [14]. An example statement is “The message tried to make a decision for me.” Total scores range from 4 to 28, mean 1–7, with higher scores indicating the message has caused a greater threat to freedom. This scale had excellent reliability α = .91.

#### Anger

Anger was measured using a four-item scale [34]. Participants were presented with a series of statements and asked to respond in accordance with how the newspaper article made them feel. An example item is “I felt angry while reading the article.” Higher scores (range 4–28, mean 1–7) represent greater anger in response to the message. To reduce the potential impact of negative priming, an additional, three-item positive emotions scale, designed by the same authors was interspersed within this measure. Scale items were, therefore, categorized as positive cognitions (happy, content, and cheerful) and anger (angry, irritated, annoyed, and aggravated). Only the four-item anger scale was used in the analysis. The anger scale demonstrated excellent reliability α = .91.

#### Counter-arguing

To measure counter-arguing in response to the message, participants completed a four-item measure adapted from previous research [35]. An example item is “I found myself actively disagreeing with the content of the article.” Higher scores are indicative of greater counter-arguing in response to the message (scoring range 4–28, mean 1–7). The scale had acceptable internal consistency α = .75.

### Readability and Credibility Measures

#### Readability

Readability was assessed with a three-item scale used previously within existing framing literature [29]. The scale demonstrated good reliability (α = .84). Participants were presented with the sentence stem “How easy or difficult was the article to…” and asked to respond using the following criteria (*read, understand, and remember*). Scores were measured on a seven-point scale from 1 (*very difficult) to* 7 (*very easy*) with higher average scores (total score range 3–21, mean 1–7) indicative of greater message readability.

#### Credibility

A three-item scale was used to measure message credibility [36]. Participants were presented with the statement “The article was…” and asked to score the message on the following adjectives (*Accurate, Authentic, and Believable*). Responses were scored on a seven-point scale from 1 (*strongly disagree) to* 7 (*strongly agree*). Higher scores (total score range 3–21, mean 1–7) represent greater message credibility. The scale demonstrated good internal consistency (α = .81).

### Statistical Analysis

The data were analyzed using IBM SPSS 25. Initially, one-way analyses of variance (ANOVAs) and chi-square tests were conducted to assess demographic differences across the experimental conditions. To test the primary hypothesis and examine whether donor intentions differed over time as a function of the experimental conditions, a 2 × 4 mixed-measures ANOVA was conducted with time (baseline and postmessage exposure) as the within-subjects factor and condition (1, 2, 3, and 4) as the between-subjects factor. Differences in preintentions and postintentions across the four experimental conditions were then explored using simple main effects. To test our second hypothesis and investigate whether components of the experimental manipulation (high- vs. low-threat language and loss- vs. gain-frame messaging) induced reactance, a series of 2 × 2 ANOVAs were conducted. To test the third hypothesis and examine whether individuals who plan to opt-out of organ donation experience heightened reactance in response to the message conditions, a 3 × 4 multivariate analysis of variance (MANOVA) was conducted. The reactance outcome variables (threat to freedom, anger, and counter-arguing) were entered as the dependent variable, with anticipated donor choice under opt-out consent (opt-in, deemed consent, not sure, and opt-out) as the independent variable. The multivariate statistic Pillai’s trace was adopted to account for unequal sizes between the anticipated donor choice groups. Univariate ANOVAs on each of the three reactance measures were conducted and a series of Games–Howell post hoc tests were used to investigate group-level differences.

## Results

### Participant Demographic Characteristics

A total of 1,350 adults from Scotland (78.3%; *n* = 1,057) and England (21.7%; *n* = 293) participated in this study. Participants’ mean age was 36.52 years, standard deviation [*SD*] = 13.55; range = 18–95. The majority of participants 78.7% (*n* = 1,063) were female and registered organ donors (71.6%; *n* = 967). Demographic information is provided in Table 1. Additional donor characteristics, including current donor status, anticipated donor status under opt-out, and awareness of the change in legislation are available as Supplementary Information 3.

**Table 1.** Participants demographic characteristics

	Experimental condition			
	1: Low threat × Gain frame (*n* = 335)	2: High threat × Gain frame (*n* = 336)	3: Low threat × Loss frame (*n* = 342)	4: High threat × Loss frame (*n* = 337)
Age, *M* (*SD*)	36.65 (13.63)	35.89 (13.41)	36.66 (13.89)	36.87 (13.29)
Gender, *n* (%)				
Female	263 (78.74%)	266 (79.17%)	274 (80.59%)	260 (77.38%)
Male	69 (20.66%)	69 (20.53%)	60 (17.65%)	69 (20.53%)
Non-binary	1 (0.30%)	0	3 (0.88%)	6 (1.79%)
Other^a^	1 (0.30%)	1 (0.30%)	3 (0.88%)	1 (0.30%)
Education, *n* (%)				
Lower education	119 (35.52%)	128 (38.21%)	121 (35.38%)	126 (37.39%)
Higher education^b^	216 (64.47%)	207 (61.79%)	221 (64.62%)	211 (62.61%)
Religious beliefs, *n* (%)				
No religion	241 (71.94%)	207 (61.61%)	210 (61.40%)	193 (57.27%)
Christian	76 (22.69%)	103 (30.65%)	111 (32.46%)	113 (33.53%)
Buddhist	0	2 (0.60%)	3 (0.88%)	3 (0.89%)
Hindu	1 (0.30%)	2 (0.60%)	1 (0.29%)	3 (0.89%)
Muslim	2 (0.60%)	3 (0.89%)	1 (0.29%)	3 (0.89%)
Jewish	1 (0.30%)	2 (0.60%)	3 (0.88%)	2 (0.59%)
Prefer to self-describe	11 (3.28%)	14 (4.16%)	13 (3.80%)	12 (3.56%)
Prefer not to say	4 (1.19%)	6 (1.79%)	3 (0.88%)	9 (2.67%)
Ethnicity, *n* (%)				
White	316 (94.33%)	320 (95.24%)	330 (96.77%)	319 (94.66%)
Asian or Asian British	8 (2.39%)	5 (1.49%)	3 (0.88%)	8 (2.37%)
Black, African or Caribbean	2 (0.60%)	2 (0.60%)	2 (0.59%)	2 (0.59%)
Mixed/multiple ethnic groups	7 (2.09%)	3 (0.89%)	2 (0.59%)	1 (0.30%)
Hispanic or Latino	0	1 (0.30%)	1 (0.29%)	5 (1.48%)
Prefer not to say/other	2 (0.60%)	5 (1.49%)	3 (0.88%)	2 (0.59%)

*SD* standard deviation.

^a^Four participants preferred not to state their gender, the remaining two identified as female to male transgender and genderqueer.

^b^Higher education was categorized as completion of a bachelor’s degree.

### Demographic Comparisons

A series of one-way ANOVAs and chi-squared tests were conducted to investigate differences in participants’ baseline donor intentions, current donor registration status, and anticipated donor choice under opt-out consent (opt-in, deemed consent, not sure, and opt-out) between the four experimental arms: no significant differences were found, *p* > .05. With regards to demographic characteristics, no significant differences in age, gender, education, ethnicity, or political, or religious beliefs were found (*p* = .80, *p* = .74, *p* = .84, *p* = .39, *p* = .96, *p* = .06, respectively).

### Planned Donor Decisions Following the Introduction of Opt-Out Consent

Frequency counts were conducted to assess anticipated donor choice under the upcoming opt-out system. Most respondents plan to opt-in, 75.6% (*n* = 1,021). Four response options were combined to represent the opt-in group: (a) participants who had formerly completed the opt-in process and plan to uphold this decision (*n* = 802), (b) individuals who had completed the opt-in process and plan to repeat this after the legislative change to reaffirm their views (*n* = 155), (c) participants who had not yet registered and plan to opt-in (*n* = 25), and, lastly, those who were unsure if they were registered and plan to opt-in (*n* = 39). In total, 13.4% (*n* = 181) of participants plan to take no action and follow deemed consent, 3.9% (*n* = 52) plan to opt-out, and 7% (*n* = 94) were unsure of their donor decision.

### Hypothesis 1: The Effect of Language Threat and Message Framing on Donor Intentions

**Fig. 4.**
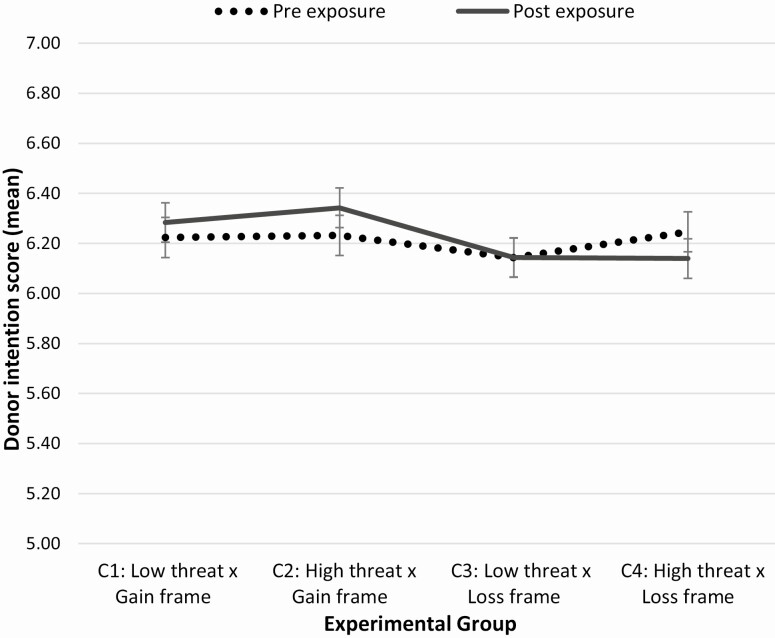
Preintention and postintention scores for the four experimental groups. Error bars represent standard error. There was no significant main effect of group or time but a significant Group × Time interaction was found. NB: The y-axis has been inflated in order to better illustrate the group by time interaction.

To test for differences in donor intentions (pre and post) as a function of the four conditions, a 2 × 4 mixed-measures ANOVA was conducted. There was no significant main effect of group: *F*(3, 1,346) = .726, *p* = .54, partial η ^2^ = .002; or time: *F*(1, 1,346) = .408, *p* =.52, partial η ^2^ =.000. However, a significant Group × Time interaction was observed, *F*(3, 1,346) = 3.57, *p* = .01. partial η ^2^ =.01. Changes in intention over time and condition are displayed in Fig. 4.

The Group × Time interaction was then explored using simple main effects. This revealed differences in intention scores over time for participants exposed to Condition 2 and Condition 4. For participants who received Condition 2 (High threat × Gain frame) intention significantly increased between baseline (*M* = 6.23, *SE =* .08) and postmessage exposure (*M* = 6.34, standard error [*SE*] = .79), *F*(1,336) = 5.00, *p* = .03, partial η ^2^ = .015. For participants exposed to Condition 4 (High threat × Loss frame), donor intentions significantly decreased between baseline (*M* = 6.25, *SE =* .08) and postmessage exposure (*M* = 6.14, *SE =* .79), *F*(1, 336) = 4.22, *p* = .04, partial η ^2^ = .012. No differences in donor intentions were found for participants exposed to Condition 1 (Low threat × Gain frame) or Condition 3 (Low threat × Loss frame).

### Hypothesis 2: The Effect of Language and Message Framing on Measures of Reactance

To assess whether components of the language and framing manipulation influenced perceptions of threat to freedom, anger, and counter-arguing, a series of two-way between-group ANOVAs were conducted. Accordingly, the four conditions were grouped in relation to their respective language threat level (Conditions 1 and 3: low threat) and (Conditions 2 and 4: high threat) and framing variants (Conditions 1 and 2: gain frame) and (Conditions 3 and 4: loss frame). For all three reactance outcome measures, Levene’s test indicated that homogeneity of variances was met, *p* > .05. Table 2 provides the means and *SD*s of threat to freedom, anger, and counter-arguing scores for each condition.

**Table 2.** Means and standard deviations (*SD*s) for reactance, credibility, and readability components across each condition

	1: Low threat × Gain frame (*n* = 335)	2: High threat × Gain frame (*n* = 336)	3: Low threat × Loss frame (*n* = 342)	4: High threat × Loss frame (*n* = 337)
Threat to freedom, *M (SD)*	2.03 (1.28)	2.25 (1.33)	2.15 (1.31)	2.28 (1.36)
Anger, *M (SD)*	1.81 (1.10)	1.82 (1.16)	2.00 (1.22)	1.98 (1.22)
Counter-arguing, *M (SD)*	2.44 (1.00)	2.41 (1.07)	2.53 (1.07)	2.52 (1.10)
Message readability, *M (SD)*	6.16 (.85)	6.11 (.92)	6.19 (.76)	6.06 (.84)
Message credibility, *M (SD)*	5.71 (.90)	5.57 (.90)	5.60 (.94)	5.48 (.94)

#### Threat to freedom

No interaction was found between threat level and framing on threat to freedom scores, *F*(1, 1,346) = .37, *ns*. There was a significant main effect of threat level, in that participants reported significantly higher threat to freedom scores in response to the high-threat language conditions (*M* = 2.26, *SD* = 1.34) in comparison to those who received the low-threat conditions (*M* =2.09, *SD* = 1.29), *F*(1, 1,346) = 5.68, *p* = .02, partial η ^2^ = .004.

#### Anger

No significant interaction effect was found between threat level and framing on anger scores, *F*(1, 1,334) = .04, *ns*. A significant main effect of message framing was found. This indicated that participants who received the loss-frame conditions reported significantly higher anger scores (*M* = 1.99, *SD* = 1.22) in comparison to those who received the gain-frame conditions (*M* = 1.82, *SD* = 1.13), *F*(1, 1,334) = 7.01, *p* < .01 partial η ^2^ = .01.

#### Counter-arguing

No significant interaction effect was found between threat level and framing on counter-arguing scores, *F*(1, 1,306) = .03, *ns*. There was also no significant main effect of threat level or framing manipulation.

### Message Readability and Credibility

To investigate differences in message readability and credibility, a series of two-way between-group ANOVAs were conducted. As described above, the four conditions were grouped in relation to their respective language threat and framing manipulation. Means and *SD*s of credibility and readability scores are provided in Table 2.

#### Readability

No interaction effect was found between language threat level and message framing on readability scores, *F*(1, 1,285) = .71, *ns*. There was also no main effect of threat level or framing manipulation. On average, the conditions were considered *easy* to read (*M* = 6.13, *SD* = 0.84).

#### Credibility

No interaction between language threat level and framing on message credibility was found, *F*(1, 1,285) = .03, *ns.* There was a significant main effect of threat manipulation in that participants exposed to the high-threat conditions reported the message to be significantly less credible (*M* = 5.52, *SD* = .92) than those who received the low-threat conditions (*M* = 5.65, *SD* = .92), *F*(1, 1,285) = 6.5, *p* = .01, partial η ^2^ = .01

### Hypothesis 3: Reactance in Individuals Who Plan to Opt-Out of Organ Donation

**Fig. 5.**
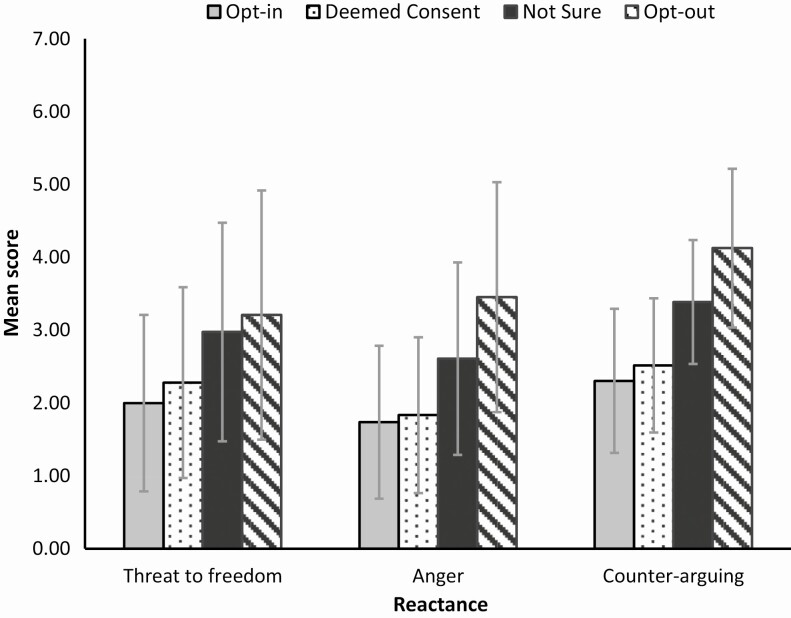
Mean reactance scores across the four donor response groups (error bars represent standard deviation). Scores across all postintervention reactance measures were significantly higher in the opt-out group.

To test whether people who plan to opt-out demonstrate heightened reactance in response to the message conditions, a 4 × 3 MANOVA was conducted. Planned donor choice under opt-out consent (opt-in, deemed consent, unsure, and opt-out) was entered as the independent variable and, in line with reactance literature, threat to freedom, anger, and counter-arguing scores were entered as dependent variables. A significant difference was found between the groups on the combined dependent variables *F*(9, 3,912) = 28.35, *p* < .001; Pillai’s *V* = .184, partial η ^2^ = .061. Mean reactance scores across the groups are graphically represented in Fig. 5. Means and *SD*s for each donor choice are available in Table 3.

**Table 3.** Means and standard deviations (*SD*s) for psychological reactance measures across the four planned donor choice groups

	Opt-in(*n* = 993)	Deemed consent (*n* = 175)	Not sure (*n* = 91)	Opt-out (*n* = 49)
Treat to freedom, *M (SD)*	2.00 (1.21)	2.28 (1.31)	2.98 (1.50)	3.21 (1.71)
Anger, *M (SD)*	1.74 (1.05)	1.83 (1.07)	2.61 (1.32)	3.45 (1.58)
Counter-arguing, *M (SD)*	2.30 (0.99)	2.52 (0.92)	3.39 (0.85)	4.13 (1.09)

Follow-up univariate ANOVAs were conducted to determine the contributing role of each reactance component. Group-level differences between each donor choice were examined using a Games–Howell post hoc test used to correct a violation of homogeneity of variances within two of the dependent variables.

#### Threat to freedom

A significant difference in threat to freedom scores between the donor groups was found, *F* (3, 1,344) = 30.88, *p* < .001; partial η ^2^ = .06. Post hoc analysis revealed that participants who plan to opt-out and those who are unsure of their planned donor decision reported significantly higher threat to freedom scores in response to the message than both those who plan to opt-in and participants who plan to follow deemed consent at *p* < .01. No difference in scores between the opt-out and not-sure groups were found. Higher scores indicate the message to have evoked a greater sense of threat to one’s freedom.

#### Anger

A one-way ANOVA revealed significant differences in anger scores between the four donor groups *F* (3, 1,332) = 52.55, *p* < .001; partial η ^2^ = .106. Post hoc exploration indicated significant differences across all group comparisons. Both respondents who plan to opt-out and those who are not sure reported significantly higher anger in response to the message than people who plan to opt-in or follow deemed consent at *p* < .001. In addition, the opt-out group also reported significantly higher anger scores than those in the not-sure group, *p* = .01.

#### Counter-arguing

Significant differences in counter-arguing scores were found between the donor groups *F* (3, 1,304) = 84.33, *p* < .001; partial η ^2^ = .162. Post hoc analysis revealed differences across all group comparisons. Highest counter-arguing in response to the message was found in respondents who plan to opt-out in comparison to those who plan to actively opt-in and follow deemed consent and those who are unsure. These differences were significant at *p* < .001.

### Supplementary Analysis: Reactance and Awareness of Opt-Out Legislation

As almost half of our sample (*n* = 622) were unaware of the legislative change, a supplementary one-way MANOVA was conducted to investigate differences in measures of reactance based on respondents’ self-reported awareness of the move to opt-out legislation. Awareness of the legislative change (yes and no/unsure) was entered as the independent variable, with threat to freedom, anger, and counter-arguing entered as the dependent variables to represent reactance. A significant difference between the groups on the combined dependent variables was observed *F* (3, 1,306) = 3.96, *p* = .008; Pillai’s *V* = .009, partial η ^2^ = .009. Univariate ANOVAs revealed significantly higher scores within each reactance component for individuals who reported being either, not aware or unsure of the legislative change. Mean reactance scores across the groups are available in Table 4.

**Table 4.** Mean reactance scores across participants self-reported awareness of opt-out legislation

	Awareness of legislation		
	Yes (*n* = 688)	No/unsure (*n* = 622)	Results
Treat to freedom, *M (SD)*	2.05 (1.27)	2.26 (1.34)	*F*(1, 1,308) = 8.46, *p* = .004, partial η ^2^ = .009
Anger, *M (SD)*	1.79 (1.13)	1.98 (1.20)	*F*(1, 1,308) = 8.83, *p* = .003, partial η ^2^ = .009
Counter-arguing, *M (SD)*	2.40 (1.08)	2.57 (1.03)	*F*(1, 1,308) = 7.94, *p* = .005, partial η ^2^ = .009

## Discussion

Existing health communication literature in relation to organ donation has predominantly focused on evaluating the utility of message variants and framing manipulations within appeals designed to increase the number of organ donor registrants [21, 22, 30]. However, nations across the world are now implementing a policy change in donor legislation and introducing opt-out consent. Thus, the act of registering as an organ donor is no longer essential in nations with opt-out systems to indicate consent for organ donation as, accordingly, all eligible adults will be automatically considered to have agreed to be a potential organ donor in the event of their death. If an individual does not want to be an organ donor, they should record this by opting out of organ donation.

In the UK, opt-out legislation is currently operational in Wales and England and is, following postponement due to the COVID-19 pandemic, scheduled for implementation in Scotland in March 2021. In line with this substantial change in policy, national awareness campaigns are ongoing to ensure widespread public awareness of opt-out consent. Although evidence demonstrates the content of organ donation campaigns to play an important role in donor attitudes, intentions, and decision-making, there is currently no research investigating this in the context of opt-out legislation. This study, therefore, builds on the existing opt-in evidence base by exploring the contribution of language and framing manipulations within communication campaigns that describe the move to opt-out consent on both, intention to donate organs and on the development of psychological reactance.

### The Effect of Language Threat and Message Framing on Donor Intentions

Within the current study, the impact of two message features, language threat level and message framing, were explored. It was predicted that the organ donation campaign containing high-threat language and loss-frame components would act to reduce intention to donate organs in comparison to messages containing low-threat, autonomy-supportive language and gain-frame components. The findings provide support for this hypothesis, in that the application of freedom-threatening language and loss-frame components significantly reduced organ donor intentions. These findings are consistent with existing health communication research, which reports the use of high-controlling language within promotional health messages to decrease intentions toward the advocated behavior [14, 17].

Notably, these results highlight the particularly potent combination of high-threat language and loss-frame components on behavioral intentions to donate organs. Indeed, exploration of the language and framing manipulations and their impact on participants’ responses to the message revealed that the two high-threat language conditions induced significantly higher perceptions of threat to freedom than the organ donation messages that used low-threat language. With regards to framing, participants exposed to the loss-frame manipulation, which detailed the lives lost annually as a consequence of the donor shortage, reported significantly higher levels of anger in response to the message compared to those who received the gain-frame manipulation. Therefore, this may have induced an unintended negative emotional response. Existing evidence offers support for this interpretation, reporting loss-frame messages to evoke guilt and increased perceptions of freedom threat due to their implicitly forceful nature [30, 37]. It is also interesting to note that, within this study, high-threat messages were perceived to be significantly less credible than low-threat, autonomy-supportive messages. This effect has also been described within extant literature on physical activity campaigns, whereby the use of high-controlling language was reported to significantly lower perceptions of credibility, characterized as decreased message expertise, trustworthiness, and sociability [17]. As credibility plays an important role in message acceptance, particularly within campaigns related to health decision-making, careful consideration into the use of high-threat language is warranted [38]. Collectively, these results advocate for the avoidance of overtly high-threat language and loss-frame statements in order to minimize the number of potential opt-out registrations.

Though the high-threat and loss-frame condition acted to significantly reduce intentions to donate, the application of high-threat language coupled with gain-frame messaging was found to significantly increase intentions to donate organs. Gain-frame manipulations, which highlight the positive impact of registering as an organ donor, may, therefore, serve to buffer the negative effects of high-threat language. Further studies testing the utility of such framing manipulations are warranted. Across the aforementioned results, it is challenging to determine why the low-threat autonomy-supportive messages had no impact on donor intentions. There is some evidence to suggest that low-threat messages can be interpreted as somewhat ambiguous and difficult to understand in comparison to more explicit messages that use high-threat or high-controlling language [17]. Therefore, a plausible explanation for this finding may be that the low-threat messages were comparatively unclear. Future research is required to investigate this.

In line with this, it is important to acknowledge that the degree of threat manipulation employed in the present study was relatively subtle in comparison to existing health communication research [14, 22]. The following excerpts depict high-threat language that successfully induced perceptions of threat to freedom within experimental manipulations. In both examples, the use of language could be considered as overtly authoritarian and somewhat accusatory; “No other conclusion makes any sense. Stop the denial. There is a problem and you have to be part of the solution.” and “Stop the denial! Given the need for organ donors, a reasonable person would consent to be an organ donor” [14, 22]. The application of such language would not be appropriate for routine use in public health campaigns as they run the risk of eliciting reactance. Comparatively, the high-threat manipulation within the current study was developed in accordance with the language used in existing organ donation press releases, for example, “Don’t want your organs to be donated? You WILL have to opt-out as ministers back law change to help transplant patients” https://www.dailymail.co.uk/news/article-5428997/You-opt-ministers-law-change.html. This has important implications for health communication literature and indicates that subtle manipulations in language, designed in line with existing communication campaigns used within the public domain, has the potential to induce a freedom threat and psychological reactance.

### Reactance in Individuals Who Plan to Opt-Out of Organ Donation

The findings also demonstrate that participants who plan to opt-out, and those who are unsure of their anticipated donor decision, are at risk of experiencing a heightened reactant response to opt-out organ donation campaigns. In sum, both groups appraised the opt-out campaigns to cause a significantly greater threat to their freedom and reported heightened anger and counter-arguing than those who plan to donate their organs either by actively opting in to organ donation or via deemed consent. These results reinforce and triangulate the findings of recent qualitative research, whereby participants perceived the upcoming legislative change as a threat to their freedom and a coercive method of procuring consent for organ donation: “if it’s opt-out you remove their choice and their voice” [10].

### Restoration of Freedom

In acknowledgement of the potentially harmful impact of reactance within communication campaigns, a number of studies have explored the utility of restoration postscript messages as a method of freedom restoration [17, 37, 39, 40]. In short, restoration postscripts are designed to mitigate the effects of reactance by reaffirming the reader’s autonomy following exposure to a freedom-threatening message [17]. Within the context of the current study, an appropriate restoration postscript would be to emphasize that readers still have a choice regarding the decision to be or not to be an organ donor. However, research examining restoration postscripts within health communication literature has reported inconsistent results. For example, though the application of restoration postscript messages within exercise campaigns was found to reduce perceptions of threat to freedom [17], no such effect was reported when applying postscript messages to campaigns promoting organ donation registration [37]. In the context of recycling communication campaigns, restoration postscripts have been effective at reducing reactance, increasing positive attitudes, and behavioral intentions [39]. Notably, this effect was only evident within the high-threat language condition. Thus, the inclusion of a restoration postscript message may present a relatively straightforward method of reducing reactance and increasing behavioral intentions within campaigns for opt-out consent.

### Implications and Future Directions

This study strengthens the existing evidence base on the impact of reactance within health-related decision-making. The transition to opt-out consent in the UK represents an extensive overhaul in organ donation policy, as such, sustained communication campaigns are essential to ensure widespread public awareness and understanding of the legislative change. Investigating the most effective way of communicating this change to the public is critical to mitigate reactance and promote informed donor decision-making. To that end, this study demonstrates the detrimental impact of a relatively subtle language and framing manipulation applied to opt-out organ donation messages on perceived threat to freedom, anger, credibility, and donor intentions. Furthermore, collaboration with UK newspapers and news sources is important to ensure that sensationalist representations of opt-out consent are avoided, and the legislative change is communicated in an appropriate way. This may be achieved through engagement between the government and the press to facilitate the development of specific practices to apply when communicating this sensitive legislative change. In line with this, future studies examining existing newspaper articles describing the transition to opt-out consent and their role in eliciting reactance-based responses is warranted. Existing literature has also endorsed the use of restoration postscript messages as a method of alleviating psychological reactance and/or increasing behavioral intentions [17, 39, 40]. Future studies examining these approaches within the context of opt-out organ donation campaigns are required.

### Limitations

A number of limitations should be considered. First, the newspaper conditions were designed by the authors and, though informed by existing organ donation press releases, this may, to some degree, limit the ecological validity of the study. However, we believe that designing the messages to emulate a newspaper article, rather than presenting print messages, may go some way toward mitigating this. Moreover, this enabled the examination of multiple message variants. The inclusion of a control condition, which only provided a description of different types of donation systems (opt-in and opt-out) may also have been helpful to determine whether reactance arose as a consequence of the experimental manipulation or simply in response to information regarding the move to an opt-out donor system. A further limitation concerns the second framing manipulation applied to the description of the Welsh opt-out consent system. Accordingly, in the loss-frame manipulation, opt-out consent was described to have “a small increase in the number of transplants” and the gain frame, “a promising increase in the number of transplants.” It may be argued that this does not align fully with traditional loss-framing interventions whereby readers are presented with overtly negative outcomes, for example, “400 people died waiting for a lifesaving transplant.” The intervention we employed could be classified as more of a neutral framing manipulation. However, this decision was made in accordance with existing descriptors of opt-out consent that feature within the public domain. Finally, it is important to also note that a large proportion of our respondents were university educated, white, females. As such, this may limit the generalizability of the study findings.

## Conclusion

In opt-out organ donation campaigns, our results demonstrate the detrimental impact of high-threat language and loss-frame messaging on donor intentions. Emphasizing the benefits of organ donation via gain-frame messaging within campaigns that use high-threat language acted to increased intentions toward organ donation. Further studies are required to examine if this is a robust and replicable finding. If it is, this type of message framing could be considered for use in future organ donation public health campaigns. In sum, careful consideration into the way this sensitive legislative change is communicated is imperative to minimize perceived threats to freedom, anger and to reduce the number of opt-out respondents.

## Funding

This study was funded by a PhD studentship awarded to Jordan Miller from the University of Stirling.

## Compliance With Ethical Standards

**Authors’ Statement of Conflict of Interest and Adherence to Ethical Standards:** All authors declare no conflicts of interest.

**Authors’ Contributions:** J.M. designed the study with considerable input and supervision from L.M., S.C., and R.O. J.M. incorporated the study into Qualtrics and monitored recruitment. J.M. analyzed the data. J.M. drafted the manuscript with significant contributions from L.M., S.C., and R.O. All authors read and approved the final manuscript. 

**Ethical Approval:** All procedures performed in studies involving human participants were in accordance with the ethical standards of the institutional and/or national research committee and with the 1964 Helsinki declaration and its later amendments or comparable ethical standards. Ethical approval for this study was obtained from the University of Stirling’s General University Ethics Panel.

**Informed Consent:** All participants provided informed consent prior to participation via an electronic selection box on the online questionnaire. The paper contains no potentially identifiable information.

The findings reported in this article have not been previously published, and the manuscript has not been submitted elsewhere.

The data from this paper have not been previously reported, and all authors confirm that we have full control of the data and agree to allow the journal to review this at any point if requested.

## Supplementary Material

kaab017_suppl_Supplementary_Information_1

kaab017_suppl_Supplementary_Information_2

kaab017_suppl_Supplementary_Information_3
