# Supplementary material for: Investigating the Effects of Threatening Language, Message Framing, and Reactance in Opt-Out Organ Donation Campaigns
Source: Ann Behav Med. 2021 May 3;56(1):50–63. doi: 10.1093/abm/kaab017 (PMC8691393; doi:10.1093/abm/kaab017)
Supplement: kaab017_suppl_Supplementary_Information_1 [file kaab017_suppl_supplementary_information_1.docx]

**Supplementary Information 1:** Study procedure diagram

**Baseline Assessment**

- (Baseline) Donor intentions
- Awareness of opt-out
- Current donor status
- Anticipated donor decision (under opt-out consent)

**Demographics**

**Randomised**

**Condition 1**

Low threat x Gain frame

**Condition 2**

High threat x Gain frame

**Condition 3**

Low threat x Loss frame

**Condition 4**

High threat x Loss frame

***Primary Outcome Measures***

| - (Post) Donor intentions - Reactance measures |
| --- |
| Threat to freedom  Anger  Counter-arguing |

Repeat exposure to allocated newspaper stimuli only for completion of readability and credibility measures

***Secondary Outcome Measures***

- Message readability
- Message credibility
