# Supplementary material for: Investigating the Effects of Threatening Language, Message Framing, and Reactance in Opt-Out Organ Donation Campaigns
Source: Ann Behav Med. 2021 May 3;56(1):50–63. doi: 10.1093/abm/kaab017 (PMC8691393; doi:10.1093/abm/kaab017)
Supplement: kaab017_suppl_Supplementary_Information_2 [file kaab017_suppl_supplementary_information_2.docx]

**Supplementary Information 2:** Language and framing manipulations used within each of the four conditions

| *Low Threat Message Components* |  | *High Threat Message Components* |
| --- | --- | --- |
| The way that people choose to register as an organ donor in Scotland and England is changing. |  | The Government have decided to change the organ donation laws in Scotland and England. |
| Currently, anyone in Scotland and England wishing to donate their organs in the event of their death can choose to opt-in and join the organ donor register. |  | Currently, anyone in Scotland and England who wants to donate their organs in the event of their death can choose to opt-in and join the organ donor register. |
| *Gain Frame OR Loss Frame Message Components*  **Gain** - Last year, 4990 people received a potentially lifesaving transplant.  OR  **Loss** - Last year, 400 people died while waiting for a potentially lifesaving transplant. | | |
| Under the new system, if an adult hasn’t registered a donor decision, they will be considered to have no objections to becoming an organ donor. This is known as deemed consent. |  | Under the Governments’ new law, adults will now be presumed to be in favour of donating their organs. This is known as presumed consent. |
| If you decide you don’t want to donate your organs you can always choose to opt-out of the donor register. |  | If you do not want to donate your organs, you must now opt-out of the donor register. |
| *Gain Frame OR Loss Frame Message Components*  **Gain** - Wales introduced a deemed consent system in 2015. The latest figures have now shown a promising increase in the number of transplants.  OR  **Loss** - The Welsh Government changed the donor law and introduced presumed consent in 2015. The latest figures have now shown a small increase in the number of transplants. | | |
| When the system changes, you have the opportunity to make the following choice:  • Opt-in if you want to donate your organs.  • Opt-out if you do not want to be a donor.  • Do nothing and you will be considered as having no objections to being a donor. |  | When the Government introduces the new law, you will now have to:  • Opt-in if you want to donate your organs.  • Opt-out if you do not want to be a donor.  • If you do nothing, it will now be assumed that you are willing to donate your organs when you die. |
