# Supplementary material for: Investigating the Effects of Threatening Language, Message Framing, and Reactance in Opt-Out Organ Donation Campaigns
Source: Ann Behav Med. 2021 May 3;56(1):50–63. doi: 10.1093/abm/kaab017 (PMC8691393; doi:10.1093/abm/kaab017)
Supplement: kaab017_suppl_Supplementary_Information_3 [file kaab017_suppl_supplementary_information_3.docx]

**Supplementary information 3:** Additional donor characteristics

|  | | | | | |
| --- | --- | --- | --- | --- | --- |
|  | Experimental Condition | | | | |
|  | 1: Low Threat x Gain Frame  (*n* = 335) | 2: High Threat x Gain Frame  (*n* = 336) | 3: Low Threat x Loss Frame  (*n* = 342) | 4: High Threat x Loss Frame  (*n* = 337) | |
| **Organ donor status** N (%) |  |  |  | |  |
| Registered donor | 238 (71.04%) | 245 (72.92%) | 237 (69.30%) | | 247 (73.29%) |
| Not registered | 61 (18.21%) | 52 (15.48%) | 68 (19.88%) | | 59 (17.51%) |
| Unsure | 35 (10.45%) | 36 (10.71%) | 36 (10.55%) | | 28 (8.31%) |
| Opted-out^a^ | 1 (0.30%) | 3 (0.89%) | 1 (0.29%) | | 3 (0.89%) |
| **Awareness of change** N (%) |  |  |  | |  |
| Yes | 190 (56.72%) | 171 (50.89%) | 171 (50.00%) | | 172 (51.04%) |
| No | 98 (29.25%) | 117 (34.82%) | 123 (35.96%) | | 129 (38.28%) |
| Not sure | 47 (14.03%) | 48 (14.28%) | 48 (14.03%) | | 36 (10.68%) |
| **Planned donor choice** N (%) |  |  |  | |  |
| Opt-in | 252 (75.22%) | 256 (76.19%) | 254 (74.27%) | | 259 (77.31%) |
| Deemed consent | 52 (15.52%) | 46 (13.69%) | 44 (12.86%) | | 39 (11.64%) |
| Not sure | 20 (5.97%) | 25 (7.44%) | 27 (7.89%) | | 22 (6.57%) |
| Opt-out | 11 (3.28%) | 9 (2.68%) | 17 (4.97%) | | 15 (4.48%) |
| ^a^ The option to record your wishes not to be an organ donor has been offered under the current opt-in system since late 2015. | | | | | |
